# Dynamic changes and driving factors of wetlands in Inner Mongolia Plateau, China

**DOI:** 10.1371/journal.pone.0221177

**Published:** 2019-08-19

**Authors:** Ying Zheng, Huamin Liu, Yi Zhuo, Zhiyong Li, Cunzhu Liang, Lixin Wang

**Affiliations:** 1 School of Ecology and Environment, Inner Mongolia University, Hohhot, China; 2 SUCCESS Centre, Inner Mongolia University, Hohhot, China; 3 Inner Mongolia Key Laboratory of Environmental Pollution Control & Waste Resource Reuse, Inner Mongolia University, Hohhot, China; CAS, CHINA

## Abstract

Wetlands are one of the most critical resources in Inner Mongolia Plateau. However, the region has experienced severe wetland loss in the context of global change. To quantify the dynamic change and the related driving forces, we extracted wetland information using multi-temporal *Landsat* images between 1993 and 2013 using ArcGIS platform and man-machine interactive interpretation. Dynamically changing characteristics for the past 20 years were analyzed, including wetland types and spatial distribution patterns of the wetlands in Inner Mongolia. We also performed correlation analysis and generalized linear models to quantify the contribution of natural and human factors to the changes in natural wetland area. Our results indicated that the total area of wetlands was 42421.2 km^2^ in 1993, and decreased to 38912.4 km^2^ in 2013, a decline ratio of 8.3%. Meanwhile, all types of wetlands showed a trend of transformation into non-wetlands. Anthropogenic factors led to the loss of natural wetlands in Inner Mongolia. In grasslands, mining coal was the dominant driver for natural wetland loss, while in arable lands, agricultural encroachment and irrigation were the primary driving forces. These findings can provide meaningful information for improving sustainable wetlands management strategies according to local conditions in different sub-regions.

## Introduction

Wetlands, the unique natural synthesis and water-land compound ecosystems, are one of the most significant ecological landscapes and human habitats on earth, playing a vital role in preventing drought and flood [1], regulating climate [2] and maintaining biodiversity [3]. Wetland ecosystems have the highest value of ecological service in all kinds of ecosystems. Globally, ecosystems provide various products and services for a total value of more than $ 33 trillion every year, of which the values contributed by costal and wetland ecosystems amounts to 15.5 trillion dollars [4]. Unfortunately, in recent decades the area of natural wetlands has remarkably shrunk, the quality has increasingly declined and ecological functions have severely degraded [5–7]. The Millennium Ecosystem Assessment reported that the decline of wetland functions significantly affects human well-beings [8]. Since the 1990s, the global wetland area has reduced by 50%, and most of the existing wetlands are fragmented [9]. Natural wetlands are continuing to decrease both in area and quality, especially in Asia [10]. As a vulnerable ecosystem, wetland is particularly sensitive to climate change and anthropogenic activities [11, 12]. The fifth assessment reports from IPCC (Intergovernmental Panel on Climate Change) predicted further impacts of global climate change, including temperature rising, precipitation regime shift, sea level rise and increasing frequency of extreme climate events [13], additionally, effective ways to control excessive human activities such as urbanization, long-term groundwater extraction, species invasion and industrial pollution are lacking [14–16]. Given these trends, firstly, the essential step is to quantify area changes of wetlands and to identify the underlying driving forces [17–19], which are indispensable for the development of sustainable management strategies.

At present, worldwide and nationwide monitoring of wetland changes based on remote sensing had been performed [10, 20, 21]. The accuracy, however, was restricted due to the larger scales. In China, a large number of studies focused on dynamic changes of wetlands and the relevant drivers at regional scales, which had confirmed the indisputable status of wetland loss and degradation, such as the Sanjiang Plain [22, 23], Qinghai-Tibet plateau [24], and coastal wetlands [25]. However, in previous studies, due to the lack of a universally accepted definition and classification of wetlands, coupled with the deficiency of long-term monitoring, the status of majority of the resources is uncertain, more accurate and detailed information at regional scale are much more indispensable.

Inner Mongolia, located in north China, which is ranking third nationally in terms of total wetland area, belongs to typical region for global change studies. Fifty wetlands have been selected to establish national nature reserves, in which Dalai Lake National Nature Reserve (Ramsar Site No.: 1146) and *Larus relictus* National Nature Reserve (Ramsar Site No.: 1148) in Ordos are listed into the List of Wetlands of International Importance. The region was selected to be the objective for the following reasons: (1) as northern ecological security barrier of China, the region has undergone severe wetland loss and degradation under the increasing resource utilization, such as coal mining, overgrazing, and intensive agricultural activities; (2) most of Inner Mongolia lies in arid and semi-arid climate zone, where the frequent droughts have a strong impact on the ecosystems in the region; (3) under the dual pressures of global climate change and extremely intensive human activities, a series of ecological problems such as grassland degradation, soil erosion, desertification and biodiversity decline in wetlands of Inner Mongolia not only seriously threaten the ecological balance of Mongolian Plateau, but also endanger ecological security of surrounding areas. At present, the region is an important source for dust storms in northern China due to degradation of wetland and grassland ecosystems [26], it is reported that millions of tons of dust from this region has transported to western Pacific regions every year, and some dust even reaches North America [27, 28]. These reasons characterize the fragile eco-environment region to be a key region for global change studies. The basic information especially dynamic change of wetlands is essential for evaluating the status of wetland resources [29], having a great impact on structure, function and ecological process of the landscape, along with species composition and biodiversity [30]. However, an overall study on wetland dynamic changes in the region has not been performed. The unclear understanding of temporal and spatial changes of wetland has also restricted local wetland conservation and management. In this paper, remote sensing images were used to investigate the dynamic of wetlands in Inner Mongolia from 1993 to 2013. Furthermore, we examined meteorological data and statistical yearbook data from the selected years to preliminarily identify the associated driving forces of natural wetland changes by statistical analysis.

## Materials and methods

### Study sites

Inner Mongolia Autonomous Region (IMAR), located in the hinterland of Eurasia (37°24′-53°22′N, 97°12′-126°04′E), covers an area of 1.18 million square kilometers. The study area is characterized by climate conditions, with a significant decrease of precipitation and a drastic increase of evaporation from east to west, crossing humid temperate, semi-humid, semi-arid, arid and extreme arid climatic zones. Meanwhile, the regional landform presents mosaic distribution of plains, mountains, plateaus and basins. Consequently, depending on the unique features of climate and landforms, Inner Mongolia has a wide variety of wetlands that are closely associated with the water source such as rivers and lakes. Besides, wetlands in the region are well developed in summer and autumn due to the synchronization of heat and rainfall [31].

### Remote sensing images and pre-processing

Wetland data set derived from multi-temporal Landsat images, including *Landsat5* TM (Thematic Mapper) images, *Landsat7* ETM+ (Enhanced Thematic Mapper Plus) images and *Landsat*8 OLI (Operational Land Imager) images from 1993 to 2013, were mainly used to assess changes of wetland area in IMAR. These images were acquired from Geospatial Data Cloud website (http://www.gscloud.cn/) and the United States Geological Survey website (http://www.usgs.gov/) with a spatial resolution of 30 m. Because of the dates of remote sensing images can directly affect wetland areas, we chose the images from June to September (in this time range, the vegetation cover is at a maximum) to ensure the consistent dates for each year. Altogether 222 *Landsat* images were collected, for which the cloud cover was less than 10%. Prior to the interpretation, all of the remote sensing images were pre-processed through standard false color composition and standard deviation stretch in order to clearly identify wetlands. The procedures were implemented in ENVI 5.1.

### Remote sensing classification system of wetland

The rationality of wetland classification system directly affects wetland identification and the interpretation precision [32]. However, currently, there is no unified criteria for wetlands classification. Some existing classification systems have been proposed on the basis of different research purposes. In this paper, a remote sensing classification system of wetland was put forward (Table 1) by making reference to the definition and classification system of wetlands at home and abroad, combining with the local conditions of wetland in Inner Mongolia and wetland interpretation in remote sensing image. Wetlands in IMAR were divided into natural and artificial wetland systems, further, natural wetland system was composed of rivers, lakes, marshes, wet meadows and salt meadows, artificial wetland was consisted of reservoirs and ponds. Accordingly, the interpretation symbols of each type of wetlands were established. More detailed information of wetland classification is exhibited in Table 1.

**Table 1:** Wetland classification system in Inner Mongolia based on remote sensing images.

Wetland systems	Wetlands categories	Characteristics
Natural wetlands	Rivers	Lands covered by rivers, includes permanent and seasonal rivers
	Lakes	Lands covered by lakes, includes permanent and seasonal lakes
	Marshes	Lands with a permanent mixture of water and woody, herbaceous or shrubby vegetations (vegetation cover ≥ 30%)
	Wet meadows	Typical grassland vegetation to marsh vegetation transition type
	Salt meadows	Meadows dominated by halophytic vegetation
Artificial wetlands	Reservoirs & Ponds	Man-made facilities for storage, electricity-generating, aquaculture and agricultural irrigation
	Canal	Man-made canal for irrigation

### Interpretation of wetland information

In our study, we adopted computer automatic extraction combined with manual interpretation to extract wetland information. Rivers, lakes and artificial wetlands were automatically extracted by calculating normalized difference water index (NDWI) (Eq 1) [33], the combination of band 2 (green) and band 4 (near-infrared) was used for *Landsat* 5 TM and *Landsat* 7 ETM+ images, while band 3 (green) and band 5 (near-infrared) were combined for *Landsat* 8 OLI images.

$$NDWI=\frac{{Band}_{green}-{Band}_{nir}}{{Band}_{green}+{Band}_{nir}}$$ (1)

Although this method could improve the efficiency of interpretation, some errors and omissions remained. Therefore, automatic extraction of wetlands needed to be supplemented by visual interpretation. In addition, the information of marshes, wet meadows and salt meadows also relied on visual interpretation. Based on field investigation points sampled by GPS, we established the relationships between each wetland type and the corresponding imagery features in terms of color, shape, location and texture, combining with the ancillary data included Google Earth images, topographic map (1:100,000) and digital elevation model (DEM, 1:100,000), the complete wetland information (with patch area > 0.01 km^2^) was finally extracted by visual interpretation.

In an accuracy test, we investigated 150 random sampling points of typical wetlands to evaluate the extraction of wetlands using confusion matrix, the result showed that the overall accuracy of wetlands extraction was high enough (90.3%), which was deemed acceptable. Then the areas of wetlands were calculated in ArcGIS 10.1 with Albers equal-area projection.

### Dynamic changes of wetlands

Dynamic features of wetlands, including dynamic degree and transition matrix, were analyzed based on ArcGIS and ENVI software by spatial statistics analysis, which contributed to quantitative evaluation of temporal change of wetlands. The transition matrix clearly detected the complexity of wetland conversions in study area. The wetland dynamic degree is calculated as:

$$K=\frac{\left(k_{b}-k_{a}\right)}{k_{a}}\times \frac{1}{T}\times 100\%$$ (2)

where *K* is dynamic degree, indicates the dynamic rate of change of wetlands in T years (%); *k*_*a*_ refers to wetland areas in the initial stage of the study (km^2^); and *k*_*b*_ refers to wetland areas in the terminal stage of the study (km^2^); T is time interval (years). Dynamic degree reflects not only changes in amplitude, but also temporal features of wetland variation [34].

### Meteorological and statistical data

Meteorological data were used to analyze general trend of climate change in IMAR was provided by Inner Mongolia Meteorological Bureau, including monthly air mean temperature and monthly precipitation. Then, annual total Thornthwaite’s potential evapotranspiration (PET) was calculated through the aforementioned meteorological data by the following steps:

$$PET=\left\{ \begin{array}{l} \;0\;T_{i}\leq 0{}^{\circ}C\\ {16d(10T_{i}/I)}^{2}\;0{}^{\circ}C<T_{i}\leq 26.5{}^{\circ}C\\ \;a_{1}+a_{2}+a_{3}T_{i}^{2}\;26.5{}^{\circ}C<T_{i} \end{array}\right.$$ (3)

where, *PET* is monthly Thornthwaite’s potential evapotranspiration; *T*_*i*_ is monthly air mean temperature; *I* is the number of days per month divided by 30; a = 6.75×10^-7^*I*^3^-7.71×10^-5^*I*^2^+1.792×10^-2^*I*+0.49239; *I* = $\sum_{i=1}^{12}i$, is annual total heating index; $i={\left|\frac{T_{m}}{5}\right|}^{1.514}$, is monthly mean heating index; *a*_*1*_ = -415.8547, *a*_*2*_ = 32.2441, *a*_*3*_ = -0.4325. We could calculate monthly PET using Eq (3), further, annual total PET is the sum of monthly PET.

We collected grazing data (total amount of livestock, including goat and sheep from 1993 to 2013), data of agricultural encroachment (area of cropland, 1993–2013), irrigation data (effective irrigation area, 1993–2013) and coal mining data (coal production, 1993–2013) from Inner Mongolia Statistical Year Books [35].

### Driving forces analysis

In order to quantify the contribution of effecting factors to natural wetland changes, the study analyzed the trends of climate change and the associated human activities from 1993 to 2013 in IMAR. We regarded annual mean temperature (AMT, in degree Celsius), annual precipitation (AP, in millimeters) and Thornthwaite’s potential evapotranspiration (PET) as the indicators of regional climate change, then grazing intensity, agricultural encroachment, irrigation and coal mining were treated as indicators of anthropogenic interventions, in which grazing intensity was characterized by the total number of goats and sheep (in million head), agricultural encroachment was indicated by the area of cultivated lands (in million hectare), irrigation was expressed by effective irrigation area (in million hectare) and mining was indicated by coal production (in Tg). Firstly, we performed correlation analysis to identify the correlativity between five selected variables and relative wetland areas (RWA, that is to say, wetland areas in 1993 were the base area of 100%, for example, the RWA of 2002 could be expressed as the ratios of areas in 2002 to 1993). Subsequently, a generalized linear model was used to calculate the contribution of each of the selected explanatory variables to natural wetland loss. We chose the relative wetland areas as dependent variables, AMT, AP, PET, grazing intensity, arable land area, effective irrigation area and coal production as independent variables. Moreover, Akaike Information Criterion (AIC) was applied to confirm the results produced from the generalized linear model, which is widely used in statistical model selection (Eq 4).

AIC=2k+nlnRSS/n (4)

Where, *k* is the number of parameters, n is the sample size. *RSS* is sum of error square. The model with the minimum AIC value will be selected as the preferred model.

Statistical analysis was performed in R 3.3.1.

## Results

### Dynamic changes in wetland in Inner Mongolia since 1990s

**Fig 1:**
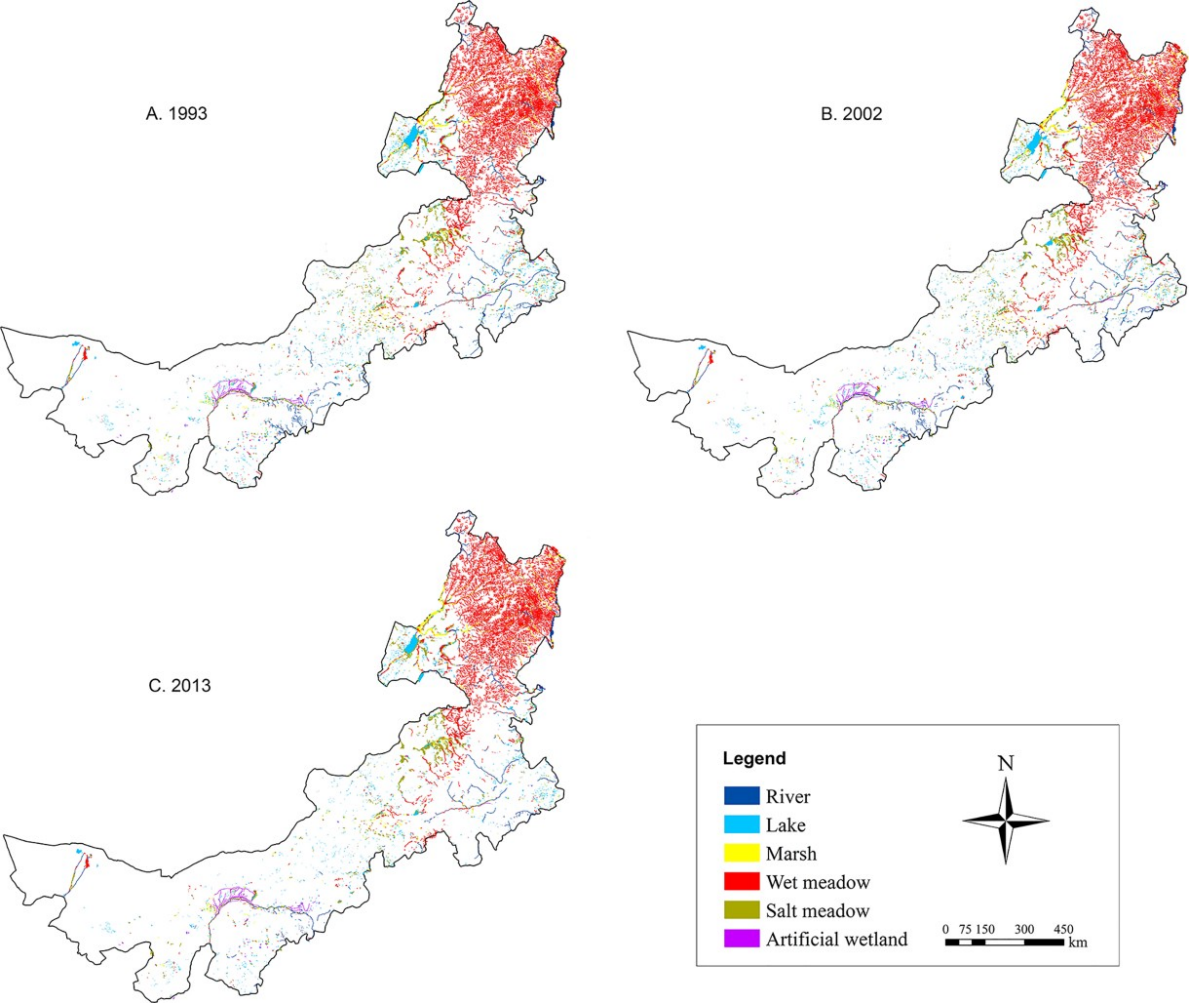
Spatial distribution of wetlands in Inner Mongolia for different stages.

**Fig 2:**
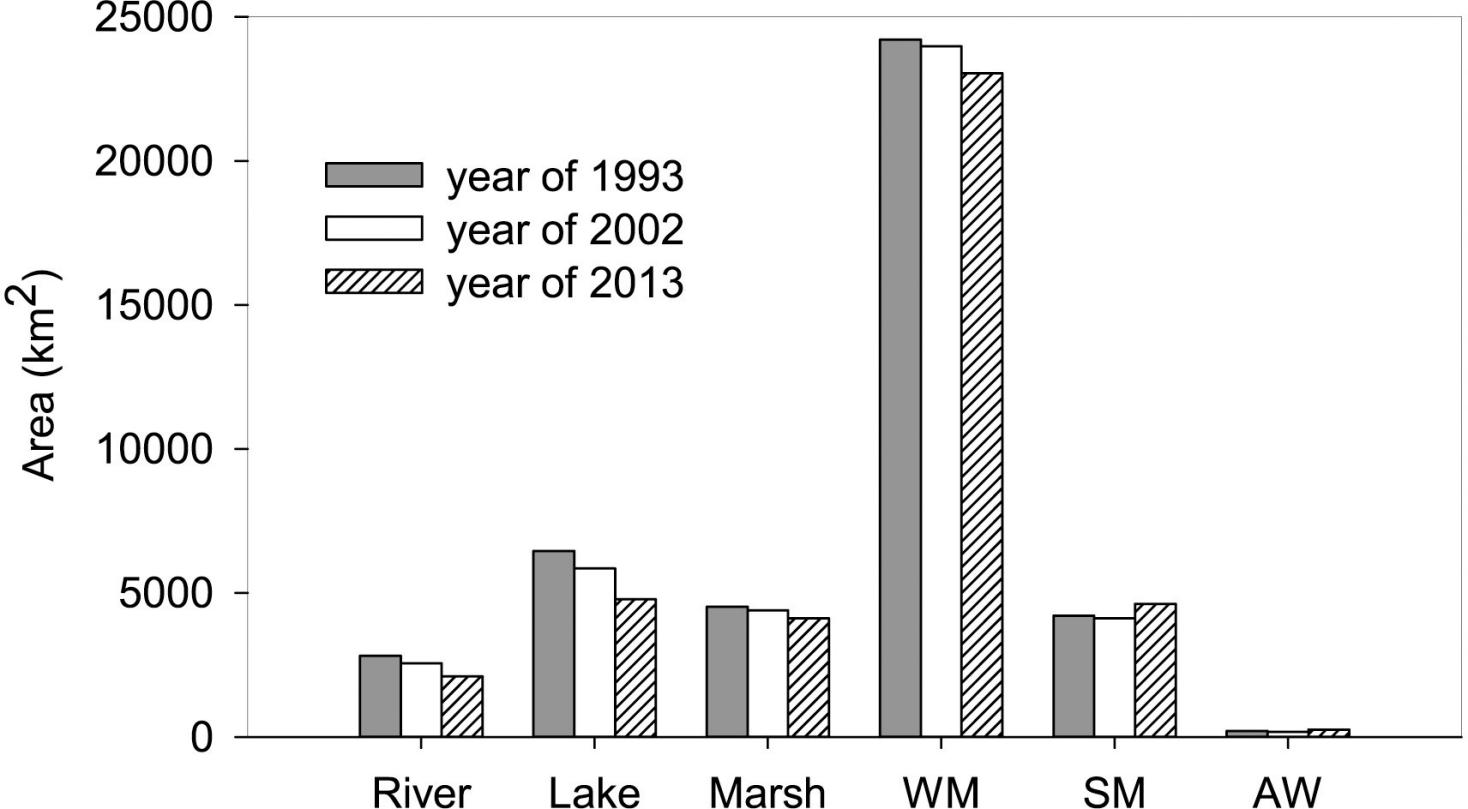
Dynamic change of wetland in IMAR. The abbreviation of wetland types: wet meadow (WM), salt meadow, artificial wetland (AW).

The spatial and temporal distribution of wetlands in Inner Mongolia is shown in Figs 1 and 2, Table 2. Spatially, wetland loss could be observed in the whole region. Temporally, the results showed that the total area of wetlands decreased from 42421.2 km^2^ in 1993 to 38912.8 km^2^ in 2013, with a decrease of 3508.4 km^2^ and the annual dynamic change rate reached -0.4%.

**Table 2:** Dynamic changes in wetland area of Inner Mongolia during 1993–2013.

Wetland types	1993 area (km^2^)	2002 area (km^2^)	2013 area (km^2^)	Changes in area (km^2^) (1993–2013)	Rate of change (%) (1993–2013)	Dynamic degree (%) (1993–2013)
River	2818.3	2555.7	2103.4	-714.9	-25.4	-1.3
Lake	6451.2	5851.8	4777.4	-1673.8	-26.0	-1.3
Marsh	4517.7	4396.7	4113.4	-404.3	-8.9	-0.5
Wet meadow	24211.5	23979.1	23044.2	-1167.3	-4.8	-0.2
Salt meadow	4210.7	4118.9	4617.7	407.0	9.7	0.5
Artificial wetland	211.7	170.1	256.7	45.0	21.3	1.1
Total areas	42421.2	41072.3	38912.8	-3508.4	-8.3	-0.4

“-” indicates the decline of wetlands

There have six major wetland types in the study region, including rivers, lakes, marshes, wet meadow, salt meadow and artificial wetlands. Wet meadows occupied the largest proportion of wetlands with an area of 24211.5 km^2^ in 1993, accounting for more than 50% of total wetland area, then they slightly decreased by 4.8% in 2013 (Table 2, Fig 2). Lakes were the second largest wetland type that covered approximately 15% of total wetland area in 1993. Since then, they had experienced rapidly shrinkage, in 2013, a number of lakes even disappeared, leaving an area of 4777.4 km^2^. Marshes decreased by 8.9% during the past decades. Rivers, as another important water source in Inner Mongolia, had a continuously decreasing trend in area from 2818.3 km^2^ to 2103.4 km^2^ during the investigated temporal extent. In contrast to the significant decline in area of above-mentioned wetland types, areas of salt meadows and artificial wetlands exhibited an increasing trend during 1993–2013, with a ratio of 9.7% and 21.3%, respectively.

### Conversion among wetland types

The transition matrix was calculated to provide information about the direction of wetland changes in IMAR, which is shown in Table 3. It can be observed that, during the study period, great changes existed between wetland types and non-wetlands. From 1993 to 2013, wetlands converted to non-wetlands primarily occurred in rivers and lakes, with a ratio of 22.65% and 16.1%, respectively, indicating severe loss of water resources in wetlands. However, non-wetlands had insignificant transformation to wetland types, which suggested that effective measures for wetland restoration in study area should be proposed. At the same time, conversions also occurred among different wetland types, in which 8.99% of lakes, 3.77% of rivers and 2.29% of marshes had converted to salt meadows. As a degraded type of wetland, salt meadows were widely distributed in the salinized area or dry lake basin. The conversion mechanism reflects that wetlands has been in a status of degradation during the past 20 years. In addition, it is worth to note that areas of artificial wetlands had obviously increased (Table 2) via the conversion from lakes (with a ratio of 0.37%), showing the evidence that constructed wetlands brought a negative effect on natural wetlands.

**Table 3:** Transition matrix of wetlands and non-wetlands in Inner Mongolia during 1993–2013 (in %).

1993	2013						
	River	Lake	Marsh	Wet meadow	Salt meadow	Artificial wetland	Non-wetland
River	70.65	1.12	0.41	1.38	3.77	0.02	22.65
Lake	0.05	69.69	1.64	3.16	8.99	0.37	16.10
Marsh	0.05	0.53	84.40	3.83	2.29	—	8.94
Wet meadow	0.23	0.21	0.46	93.87	1.06	0.00	4.17
Salt meadow	1.66	0.57	0.24	0.85	80.87	0.05	15.76
Artificial wetland	0.10	0.35	0.10	0.00	0.00	91.40	8.05
Non-wetland	0.02	0.01	0.01	0.01	0.02	—	99.93

### Driving factors in natural wetland loss

**Fig 3:**
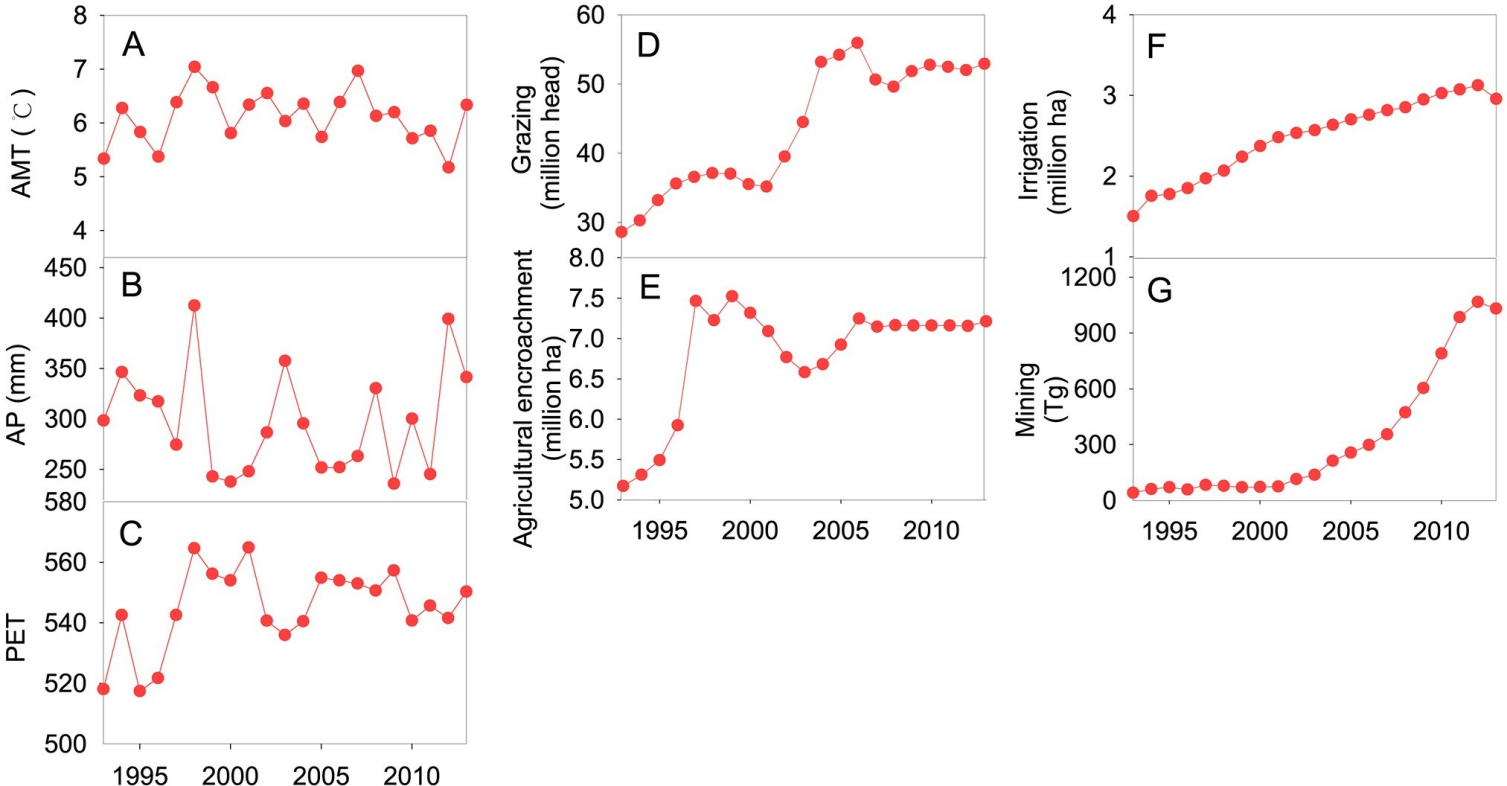
Changes of climate and human activities in Inner Mongolia during1993-2013. (A) AMT (annual mean temperature), (B) AP (annual precipitation), (C) PET (potential evapotranspiration), (D) Grazing intensity characterized by number of goats and sheep, (E) Agricultural encroachment indicated by area of cultivated lands, (F) Agricultural irrigation expressed by area of effective irrigation, (G) Mining indicated by coal production.

Over the past 20 years, PET of Inner Mongolia showed a significant increase (Fig 3C, p<0.05), which might accelerate regional aridification since 1990s and further become potential driving force for natural wetland loss. By contrast, a slight variation in AMT and AP was observed. It is obvious that anthropogenic factors of IMAR have experienced a dramatically increase, being underlying causes for natural wetland shrinkage. As shown in Fig 3D, the number of goats and sheep increased from 28.6 million heads in 1993 to 52.9 million heads in 2013, a ratio of nearly 50%. The contradiction between overgrazing and wetland protection was gradually outstanding. Excessive agricultural activities became another possible cause for wetland variation. In cropland of IMAR, the area of arable land was rapidly increased during study period (approximately 39.5%, Fig 3E), agricultural encroachment resulted in the conversion of numerous areas of natural wetlands to arable lands. On the other hand, the exploitation of water sources for agricultural irrigation directly led to wetland loss. The area of effective irrigation had increased from 1.5 million ha in 1990s to 3.1 million ha in 2010s (Fig 3F). In addition, coal mining might be treated as another great threat to wetland resources. Inner Mongolia has abundant coal resources, since early 2000s, coal mining has excessively conducted in this region, especially in grasslands, the coal production rapidly increased from 72.5Tg in 2000 to 1,030.4Tg in 2013 (Fig 3G).

Considering the fact that the dominant human activities related to natural wetland variation are significantly different in grasslands and cultivated areas, in which coal resources were mainly exploited in grasslands and agricultural activities were widely occurred in arable lands, we explored the major driving forces of natural wetland loss in grasslands and arable lands separately (Table 4). The results of correlation analysis reflected the significantly negative relationship between natural wetland areas and intensive anthropogenic activities. As the results of generalized linear model shown in Table 4, coal mining explained 62.8% (p<0.01) to natural wetland decline in grasslands, while in arable lands, agricultural encroachment and irrigation jointly contributed to natural wetland loss, 40.94% for the former (p<0.01) and 45.23% for the latter (p<0.01).

**Table 4:** Generalized linear model on the relationships between relative wetland areas and selected variables of grasslands and arable lands.

Method		Grasslands		Arable lands	
Correlation analysis	Variable	r		r	
	AMT	-0.27		-0.16	
	AP	0.11		0.01	
	PET	-0.31		-0.25	
	Agricultural irrigation	-0.12		-0.60^*^	
	Agricultural encroachment	-0.02		-0.83^**^	
	Grazing	-0.17		-0.88^***^	
	Coal mining	-0.60^*^		-0.49	
Generalized linear model	Variable	MS	SS%	MS	SS%
	AMT	121.92	9.42	105.32	2.62
	AP	9.47	0.73	300.25	7.46^*^
	PET	125.11	9.68	49.62	1.23
	Agricultural irrigation	0.51	0.04	1821.17	45.23^**^
	Agricultural encroachment	0.81	0.06	1648.69	40.94^**^
	Grazing	122.63	9.47	88.94	2.21
	Coal mining	813.23	62.85^**^	0.05	0.00
	Residuals	100.29	7.75	12.62	0.31

^*^P<0.05;

^**^P<0.01;

^***^P<0.001

Of the candidate models, the best models with minimum of AIC were selected to find the contribution of explanatory variables by generalized linear model. The results were coincided with the generalized linear model (Table 4), further indicating that the driving forces of natural wetland changes were different between grassland and arable land regions (Table 5): in grasslands, intensive coal mining was the main force, in farmlands region, agricultural encroachment and irrigation had fiercely influenced the local wetlands.

**Table 5:** Best models selected by Akaike Information Criterion and generalized linear model between wetland and selected variables by Akaike Information Criterion for different regions in IMAR.

	Grasslands		Arable lands	
Best model	Wetland ~ Mining		Wetland ~ Agricultural activities	
AIC	61.99		39.32	
Variables	MS	SS%	MS	SS%
AMT			105.32	2.69
PET			173.87	4.44^*^
Mining	569.94	84.78^*^		
Agricultural irrigation			1917.99	48.93^***^
Agricultural encroachment			1696.58	43.28^***^
Residuals	102.29	15.22	26.36	0.67

^*^P<0.05,

^***^P<0.001

## Discussions

### Dynamic change of wetlands in IMAR

Our study has detected that natural wetlands in study area has been in a state of overall contraction over the past 20 years in the context of global changes, when economic development has rapidly increased in Inner Mongolia plateau. We established wetland dynamic dataset derived from *Landsat* images based on the strict and uniform criteria in data processing in order to make sure the high accuracy of wetland interpretation. Up to now, although worldwide and national wetland inventories have been conducted [10, 21, 36, 37], it is difficult to make a comparison due to the inconsistent research purpose, wetland definition, classification system and spatial resolution of satellite data. In our study, the total areas of wetlands (with surface area > 0.01km^2^) in Inner Mongolia have declined from 42421.2 to 38912.8 km^2^ during 1993–2013, in which lakes and rivers have the greatest loss with 26.0% and 25.4%, respectively (Table 2), which reflected the severe water consumption of wetland ecosystems. The assessment of spatiotemporal changes of wetlands provides important information for further exploring the associated driving forces.

### Effects of anthropogenic activities on wetland changes

During the past 20 years, the GDP of Inner Mongolia in 2013 has been up to 300 times higher than that in 1993 [38], especially, Ordos has drastically transited from a typical poor region to the China’s 14^th^ richest city [39], which at the long-term expense of natural ecosystems. The driving mechanism of natural wetland changes in Inner Mongolia were preliminarily analyzed using natural and anthropogenic factors by statistical methods. For grassland region, natural wetland decline was primarily driven by coal mining, while in farmland region, agricultural activities included agricultural encroachment and irrigation were the major drivers.

#### Influence of agricultural activities on natural wetland shrinkage in cultivated areas

**Fig 4:**
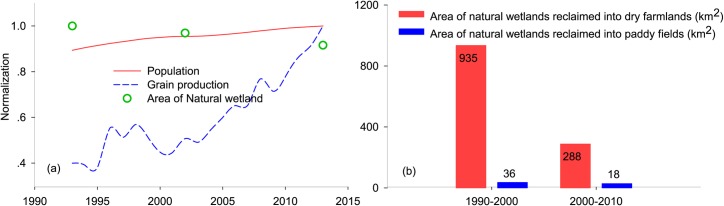
(a) Changes in normalized areas of natural wetland, population and grain production in Inner Mongolia; (b) Temporal variation of natural wetlands reclaimed into farmlands (km^2^).

Agricultural activities are certainly recognized as the leading anthropogenic drivers associated with natural wetland loss [40]. Due to underestimation of the great ecological values of wetland resources, excessive reclamation has become very common in wetlands for increasing the area of arable lands [41]. Especially in developing countries, with serious decline in South America, Southeast Asia, East Asia and Africa, where a large number of natural wetlands had been continuously converting to agricultural lands under the great pressure of the increasing population and requirement for food [17, 42–44]. The areas of natural wetland loss in Inner Mongolia caused by agricultural encroachment was second largest in China [43]. The increase in demand for food should be attributed to the rapid growth of regional population. As shown in Fig 4A, between 1993 and 2013, the tendency of population and grain production in Inner Mongolia presented a high increasing rate, 12.4% and 150%, respectively, coupled with a series of national policies in agriculture (e.g., rescission of agricultural tax) [45], which strongly promoted the large alteration of natural wetlands to cultivated lands. A total area of 1277 km^2^ natural wetlands had been replaced by farmlands, mainly occurred in southeastern and northeastern areas of Inner Mongolia [43], where located in semi-humid zones that was suitable for developing dry farming [46]. Additionally, natural wetlands that converted to farmlands in IMAR were primarily reclaimed into dry farmlands, and relatively fewer portion were reclaimed to paddy field (Fig 4B). As a result, irrigation by extracting water from rivers, lakes and groundwater for dry farmlands had become another major driver of natural wetland loss.

**Fig 5:**
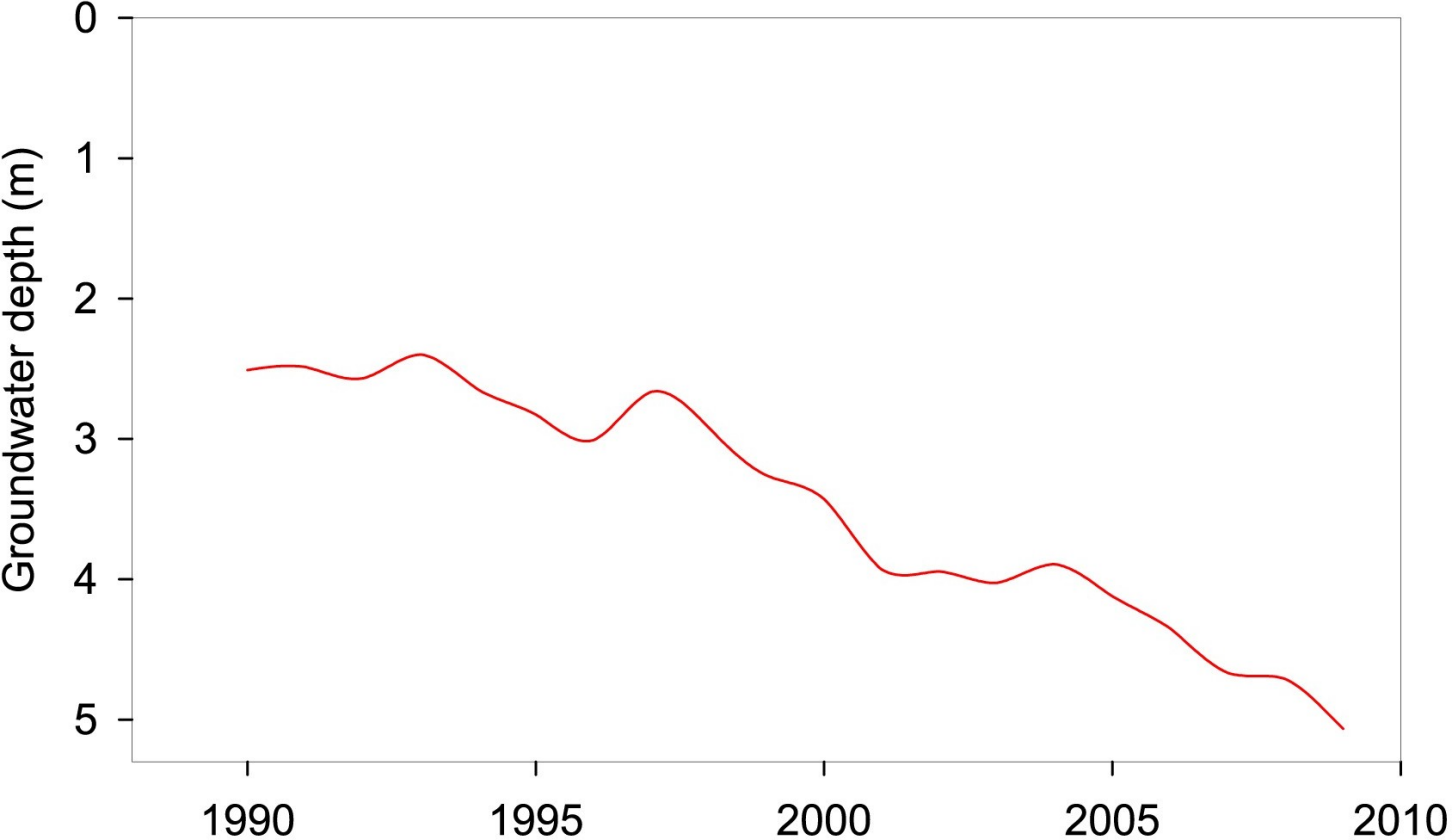
Changes in groundwater depth of Tongliao City in Inner Mongolia under the effect of agricultural irrigation.

With the planting of crops, the pumping of water resources for irrigation had cut off the rivers, e.g. Wulagai River, and shrunk, even dried up the lakes such as Dalinuoer Lake [47], Wulagaigaobi Lake [48] and Naiman Xihu [49]. According to Tao [16], in the cropland region of Inner Mongolia, irrigation explained as high as 80% of lake area shrinkage. The negative impact of irrigation on groundwater had also aroused great concern. For example, plain areas in Tongliao City (located in southeastern area of IMAR), where agricultural irrigation water accounted for 93% of total groundwater exploitation [50], and the groundwater depth had dropped from 2.5m below the soil surface to 5.2m during 1990–2009, at a rate of 0.135m yr^-1^ (Fig 5). Consequently, wetlands that were completely or partially dependent on groundwater supply had been strongly affected by the increasing groundwater depth in surface area [51].

#### Effect of coal mining on area variation of natural wetland in grassland areas

**Fig 6:**
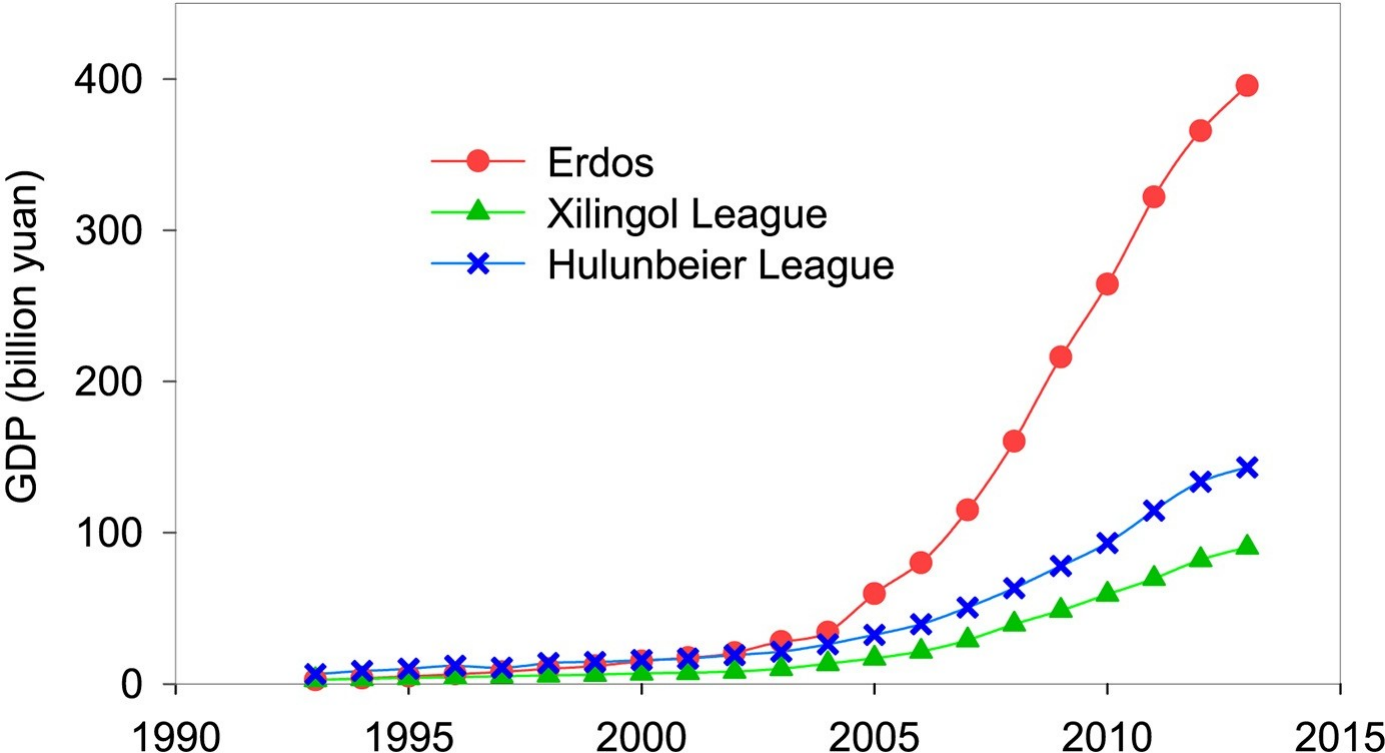
GDP of the typical coal mining areas in Inner Mongolia during the study period [38].

The grasslands of Inner Mongolia are rich in coal resources, where deposit about one-third of coal reserves in China [52], and a dramatically increasing number of enterprises had been established for coal mining. During the past years, it is no doubt that coal mining had brought high growth rate of local GDP, especially, in which GDP of Ordos in 2013 as about 200 times higher compared to that in 1993 owing to coal exploitation and export (Fig 6). However, the natural ecosystems had been adversely affected by human activities associated with coal mining in grassland regions. First, coal mining is extremely water-consumptive industry, according to estimates, 2.5 ton water had been consumed for the extraction of every ton of coal [53]. Second, coal mining had destroyed aquifer structure, then a large amount groundwater gushed into the mines, which reduced groundwater level and the supply of surface water, thus resulting in area shrinkage of surface water [54, 55]. Finally, coal mining has directly and indirectly affected wetland vegetation. On one hand, surface subsidence induced by coal mining physically damages vegetation roots, which leads to death of partial wetland vegetation [56]. An estimate reported that, in China, an average of 0.2 ha surface subsidence occurred for exploiting every 10,000 ton of coal [57], then we could extrapolate that the value was range from 800 to 20,600 ha in Inner Mongolia during the study period, which resulted in a large-scale salinization in east-central mining areas and the consequent wetland loss, accelerating soil erosion and desertification. On the other hand, the impact of coal mining on groundwater and soil water indirectly affects water absorption of vegetation and the subsequent changes in vegetation evolution and growth of wetlands [58, 59].

The major mining areas of Inner Mongolia concentrated in Ordos, Xilingol and Hulunbeier, however, coal mining had severely damaged local wetland ecosystems. Wetlands in Ordos are primarily relied on groundwater [60, 61]. The coal mining activities in Ordos began to a unprecedently large-scale expansion since 2000 in response to the China’s western development strategy [62]. During the study period, the total coal production in Ordos was 3,902 million ton [38], with an expense of more than 5,178 million m^3^ groundwater [16], consequently, many lakes and rivers were dried up, e.g. Taolimiao-Alashan Nur. In Yu Shenfu Mining Area, where located in Kuye river basin, the base flow of the river lost 2.038 m^3^ for every ton of coal extraction [63], and intensive mining contributed 71.5% for the reduction of groundwater level (more than 8m) [64].The water supply for local wetland vegetation had been significantly reduced, a large number of dominant wetland vegetation (*Carex tristachya*, *Suaeda glauca Bge*, *Phragmites australis*, *Iris ensata Thunb* and *Achnatherum splendens*) had been replaced by meso-xerophytes (*Leymus chinensis*, *Artemisia glauca* Pall and *Artemisia desterorum* Spreng) [65].

In Wulagai basin of Xilingol grasslands, four large-scale projects of coal power and chemical industry have been designed and constructed depend on rich coal reserves. Since 2004, the incoming river of wetlands in middle and lower reaches of basin had been cut off by a constructed reservoir that provides 47.6 million m^3^ of water for surrounding mining industries every year, becoming the major reason for the death of Wulagai wetlands [66]. Wetlands in Wulagai basin were completely supplied by the local drainage, a large number of lakes had disappeared due to the cutoff of river, with an increasing area of 200 km^2^ of water erosion desertification [66]. Wetland vegetation had experienced a succession to Suaeda salinity meadow and bare saline-alkaline patch [67]. The nationally important wetlands had been completely dried up in 2006 [67], which became key source region of dust storms [68].

### Legislation of wetland protection

The significant wetland loss had implied the imperfect wetland protection and management law system. At present, there are no specific laws and regulations on wetland protection and utilization in our country. The existing laws and regulations are too scattered to constitute a complete system. Adding to the lack of understanding in ecological values of wetlands, the IMAR had appeared a tendency of reduction and fragmentation in wetland area, deterioration in ecological quality and functions.

## Conclusions and perspective

Wetlands in Inner Mongolia had experienced serious changes during the past 20 years. The evolution was closely associated with human activities, while the driving forces of natural wetland change were divergent in different sub-regions: natural wetlands in grasslands decreased under the intensive human activities of coal mining, while in arable lands, natural wetland loss resulted from agricultural encroachment and irrigation. Unfortunately, if we remain insensitive to the severe situation, the risk of rapid reduction of wetlands in Inner Mongolia will be further aggravated. Therefore, the more efficient measures should be taken to contain the wetland decline induced by anthropogenic activities. Considering the different drivers in different regions, wetland management should be adjusted to local conditions. The local governments should strengthen propaganda education and improve legislation to enhance sympathies and awareness of wetland protection and management. The government of IMAR had passed “Wetland Protection Ordinance of Inner Mongolia Autonomous Region” on May 31, 2007, which would lay a foundation for further formulate and perfect the legislation so that wetlands protection will be placed on a legal track as soon as possible in study region.
